# Prevention of LPS-Induced Acute Lung Injury in Mice by Progranulin

**DOI:** 10.1155/2012/540794

**Published:** 2012-08-15

**Authors:** Zhongliang Guo, Qinchuan Li, Yang Han, Yongjie Liang, Zengguang Xu, Tao Ren

**Affiliations:** ^1^Department of Respiratory Medicine, East Hospital, Tongji University School of Medicine, 150 Jimo Road, Pudong New Area, Shanghai 200120, China; ^2^Department of Cardiothoracic Surgery, East Hospital, Tongji University School of Medicine, Shanghai 200120, China; ^3^Department of Pathology, East Hospital, Tongji University School of Medicine, Shanghai 200120, China; ^4^Department of Scientific Research, East Hospital, Tongji University School of Medicine, Shanghai 200120, China

## Abstract

The acute respiratory distress syndrome (ARDS), a clinical complication of severe acute lung injury (ALI) in humans, is a leading cause of morbidity and mortality in critically ill patients. Despite decades of research, few therapeutic strategies for clinical ARDS have emerged. Here we carefully evaluated the effect of progranulin (PGRN) in treatment of ARDS using the murine model of lipopolysaccharide (LPS)-induced ALI. We reported that administration of PGRN maintained the body weight and survival of ALI mice. We revealed that administration of PGRN significantly reduced LPS-induced pulmonary inflammation, as reflected by reductions in total cell and neutrophil counts, proinflammatory cytokines, as well as chemokines in bronchoalveolar lavage (BAL) fluid. Furthermore, administration of PGRN resulted in remarkable reversal of LPS-induced increases in lung permeability as assessed by reductions in total protein, albumin, and IgM in BAL fluid. Consistently, we revealed a significant reduction of histopathology changes of lung in mice received PGRN treatment. Finally, we showed that PGRN/TNFR2 interaction was crucial for the protective effect of PGRN on the LPS-induced ALI. Our findings strongly demonstrated that PGRN could effectively ameliorate the LPS-induced ALI in mice, suggesting a potential application for PGRN-based therapy to treat clinical ARDS.

## 1. Introduction 

The acute respiratory distress syndrome (ARDS), a clinically important complication of severe acute lung injury (ALI) in humans, is a significant cause of morbidity and mortality in critically ill patients [1–5]. Infectious etiologies, such as sepsis and pneumonia, are leading causes of ALI [1, 2, 5]. Histologically, ALI in humans is characterized by a severe acute inflammatory response in the lungs and neutrophilic alveolitis [1, 5]. The physiological hallmark of ARDS is disruption of the alveolar-capillary membrane barrier, leading to development of noncardiogenic pulmonary edema, in which a proteinaceous exudate floods the alveolar spaces, impairs gas exchange, and precipitates respiratory failure [1, 5–7]. ALI can result in persistent respiratory failure and prolonged dependence on mechanical ventilation, increasing susceptibility to multiorgan dysfunction and mortality [8]. Despite extensive investigation aimed at early diagnostic and pathogenetic factors of ALI, current management is mainly supportive, as specific therapies have not been identified [5, 9–13]. Animal models focused on ALI pathogenesis have yielded insights into mechanisms that initiate injury; however, little is known about potential determinants of resolution [8]. Thus, new strategies are still required for achieving effective treatment of ALI, which might ultimately aid the clinical therapy for ALI patients. 

Progranulin (PGRN), also known as granulin epithelin precursor (GEP), PC-cell-derived growth factor (PCDGF), proepithelin, and acrogranin, is an evolutionarily conserved, secreted glycoprotein with 7 granulin (GRN) repeats [14, 15]. PGRN played a critical role in a variety of physiologic and disease processes, including early embryogenesis, wound healing, host defense, and tumorigenesis [15–20]. Of interest, recent findings suggested that PGRN was a key regulator of inflammation and that PGRN might mediate its anti-inflammatory effects, at least in part, by blocking TNF-*α* binding to its receptors [15]. However, whether PGRN could inhibit the lung inflammation and ultimately ameliorate the ALI was still unclear. Recent evidence showed that elevated soluble tumor necrosis factor-*α* receptor levels in BAL fluid were found to be associated with poor patient outcome in ALI [21], implying that blockade of PGRN by the soluble tumor necrosis factor-*α* receptor might contribute to the development of ALI. Thus, we hypothesized that PGRN might exert as a promising molecule for treatment of inflammation in ALI. 

To address this issue, here we carefully evaluate the potential role of PGRN in treatment of ALI using the murine model of LPS-induced ALI. We found that administration of PGRN significantly reduced LPS-induced pulmonary inflammation and resulted in remarkable reversal of LPS-induced increases in lung permeability, accompanied by a significant reduction of histopathology changes of lung. Our findings strongly demonstrated that PGRN could effectively ameliorate the LPS-induced acute lung injury in mice, suggesting a potential role for PGRN-based therapy to treat clinical ARDS. 

## 2. Materials and Methods 

### 2.1. Mice

Female BALB/c mice at 6 weeks old were purchased from the Center of Experimental Animals of Tongji University. All mice were housed in the pathogen-free animal facilities of Tongji University School of Medicine. All animal experiments were performed according to the guide for the ethical guidelines of the Shanghai Medical Laboratory Animal Care and Use Committee and the ethical guidelines of the Tongji University Laboratory Animal Care and Use Committee. 

### 2.2. Murine Model of LPS-Induced ALI

The murine model of LPS-induced ALI was established as previous reported [5]. Briefly, female BALB/c mice (*n* = 6 per group) were anaesthetized and orally intubated with a sterile plastic catheter, and challenged with intratracheal instillation of 800 *μ*g of LPS (E. coli 055:B5; Sigma) dissolved in 50 *μ*L of normal PBS. Naive mice (without LPS instillation) were injected with the same volume of pyrogen-free PBS to serve as controls. Mice were humanely killed at 3 d after LPS challenge to collect tissues for analysis. TNFR1 (CD120a) and TNFR2 (CD120b) antibodies for neutralizing studies were purchased from eBioscience. Our initial experiment showed that 200 *μ*g of TNFR2 antibody was effective to significantly inhibit the protective effect of PGRN on the LPS-induced ALI. Thus, 200 *μ*g of TNFR1 antibody or TNFR2 antibody was used for neutralization experiment in this study. 

### 2.3. PGRN

Recombinant murine PGRN was purchased from R&D Systems. Groups of mice were treated with PGRN via intratracheal instillation 30 min after their challenge with LPS. The second administration of PGRN was performed 40 h after the first time. The administration dose of PGRN was 2 *μ*g per mouse which was based on our initial experiments. The level of PGRN in BAL fluid was determined by western blot using the murine PGRN affinity purified polyclonal antibody (R&D Systems) or by ELISA using the commercial murine PGRN ELISA kit (R&D Systems). 

### 2.4. Determination of Total Cells and Neutrophils

According to previously described [5], BAL was performed by instilling 0.9% NaCl containing 0.6 mmol/L ethylenediaminetetraacetic acids in two separate 0.5 mL aliquots. The fluid was recovered by gentle suction and placed on ice for immediate processing. An aliquot of the BAL fluid was processed immediately for total and differential cell counts. The remainder of the lavage fluid was centrifuged and the supernatant was removed aseptically and stored in individual aliquots at −70°C. Total cell counts in BAL fluid were determined using a haemocytometer. Number of neutrophils was calculated as the percentage of neutrophils multiplied by the total number of cells in the BAL fluid sample. All analyses were performed in a blinded fashion. 

### 2.5. Measurement of Proinflammatory Cytokines, Chemokines, Albumin and IgM

In line with previously described [5], BAL fluid collected was centrifuged at 800 g for 10 min, and supernatant was collected for analysis of total protein, albumin, IgM, and cytokine/chemokine levels. Proinflammatory cytokine levels including TNF-*α*, IL-1*β*, and IL-6 in BAL fluid were measured with murine cytokine-specific Quantikine ELISA kits (R&D Systems). Chemokine levels including Cxcl2, JE (the murine homolog of human CCL2) and KC (the murine homolog of human IL-8) in BAL fluid were measured using cytokine-specific bead kits (R&D Systems). Albumin and IgM levels in BAL fluid samples were measured using with a murine-specific albumin ELISA kit (ALPCO Diagnostics) and a murine-specific IgM ELISA kit (Bethyl Laboratories), respectively. All the measurements were performed according to the manufacturer's instructions. 

### 2.6. Histopathology

Lung tissues were fixed in 4% paraformaldehyde, embedded in paraffin, and cut into 5 *μ*m thick sections. Sections were stained with hematoxylin and eosin, and images were taken with a Nikon Eclipse E800 microscope (200x). For the lung injury score, images were evaluated by an investigator who was blinded to the identity of the slides as previously described [5, 22]. In brief, the extent of the pathological lesions was graded from 0 to 3 as shown in Table 1. The score for each animal was calculated by dividing the total score for the number of sections observed. 

### 2.7. Statistical Analysis

Differences between the treated groups versus the injured group were assessed using a one-way ANOVA with statistic software (GraphPad Prism version 4.00). A value of *P* < 0.05 was considered statistically significant.

## 3. Results 

### 3.1. PGRN was Downregulated in BAL Fluid of LPS-Induced ALI Mice

**Figure 1:**
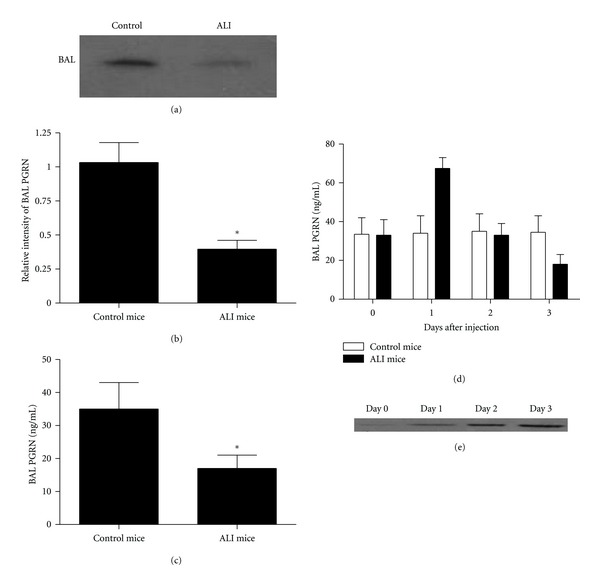
PGRN was downregulated in BAL fluid of LPS-induced ALI mice. Groups of mice were challenged with LPS for 3 days. (a) The level of PGRN in BAL fluid in LPS-induced ALI mice or control mice was determined using western blot on day 3. (b) A histogram of the relative amounts of PGRN in BAL fluid from three individual experiments was shown. (c) The level of PGRN in BAL fluid in LPS-induced ALI mice or control mice was determined using ELISA on day 3. (d) The level of PGRN in BAL fluid in LPS-induced ALI mice or control mice was determined using ELISA at the indicated time. (e) The expression of granulin in BAL fluid in LPS-induced ALI mice was detected using western blot at the indicated time. Data are represented as mean ± standard deviation of one experiment consisting of three replicates. Experiments were performed in triplicate. *P < 0.05.

To assess the potential role of PGRN in LPS-induced ALI, we determined the level of PGRN protein in bronchoalveolar lavage (BAL) fluid of LPS-induced ALI mice using western blot at day 3 after LPS challenge. We found that the level of PGRN in BAL fluid was significantly decreased on day 3 in mice challenged with LPS compared with the control groups (Figures 1(a) and 1(b), *P* < 0.05). To further confirm this result, we further performed ELISA assay to detect the level of PGRN in the BAL fluid. Similarly, we revealed that the protein level of PGRN was downregulated in BAL fluid on day 3 in LPS-induced ALI mice (Figure 1(c), *P* < 0.05). Further, we evaluated the time course of PGRN levels in BAL fluid in LPS-induced ALI mice and the control mice. As shown in the Figure 1(d), we found a substantial increase of PGRN protein on day 1 and then decreased since day 2, which indicated that PGRN might be subjected to proteolysis during inflammation in lung. Consistently, we indeed revealed an elevated expression of granulin, which were the units of PGRN, in BAL fluid in LPS-induced ALI mice (Figure 1(e)). Combing these findings indicated that PGRN might be involved in the development of ALI. 

### 3.2. PGRN Maintained the Body Weight and Survival of LPS-Induced ALI Mice

**Figure 2:**
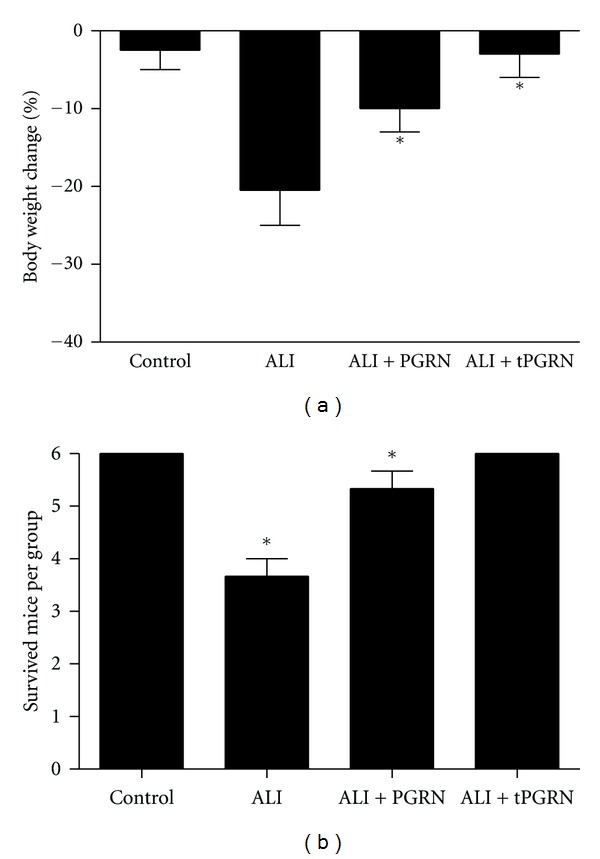
Administration of PGRN maintained the body weight and survival of ALI mice. Groups of mice were challenged with LPS and treated with PGRN 30 min later. The tPGRN represented that PGRN was administered twice at 40 h intervals. Three days after LPS challenge, the mice were assayed for their body weight relative to the baseline (a) and survival (b). Three animal experiments and each time has six animals per group were performed. *P < 0.05.

To access the potential role of PGRN in the development of ALI, we evaluated the effect of PGRN administration in the maintenance of body weight and mortality of LPS-induced ALI mice. As shown in Figure 2(a), we revealed that the loss of body weight was about 20% in LPS-induced ALI mice. Notably, we found that administration of PGRN effectively abrogated the loss of body weight of LPS-induced ALI mice (*P* < 0.05). Interestingly, when PGRN was administered twice at 40 h intervals, it could further maintained the body weight of LPS-induced ALI mice to a level similar to the control mice (Figure 2(a), *P* < 0.05). Furthermore, we found that the mortality was approximately 40% in LPS-induced ALI mice, while administration of PGRN in LPS-induced ALI mice effectively maintained their survival, which was more apparent in ALI mice received PGRN twice at 40 h intervals (Figure 2(b), *P* < 0.05). These findings suggested that PGRN was an effective candidate for preventing the development of ALI. 

### 3.3. PGRN Attenuated the Acute LPS-Induced Pulmonary Inflammation

**Figure 3:**
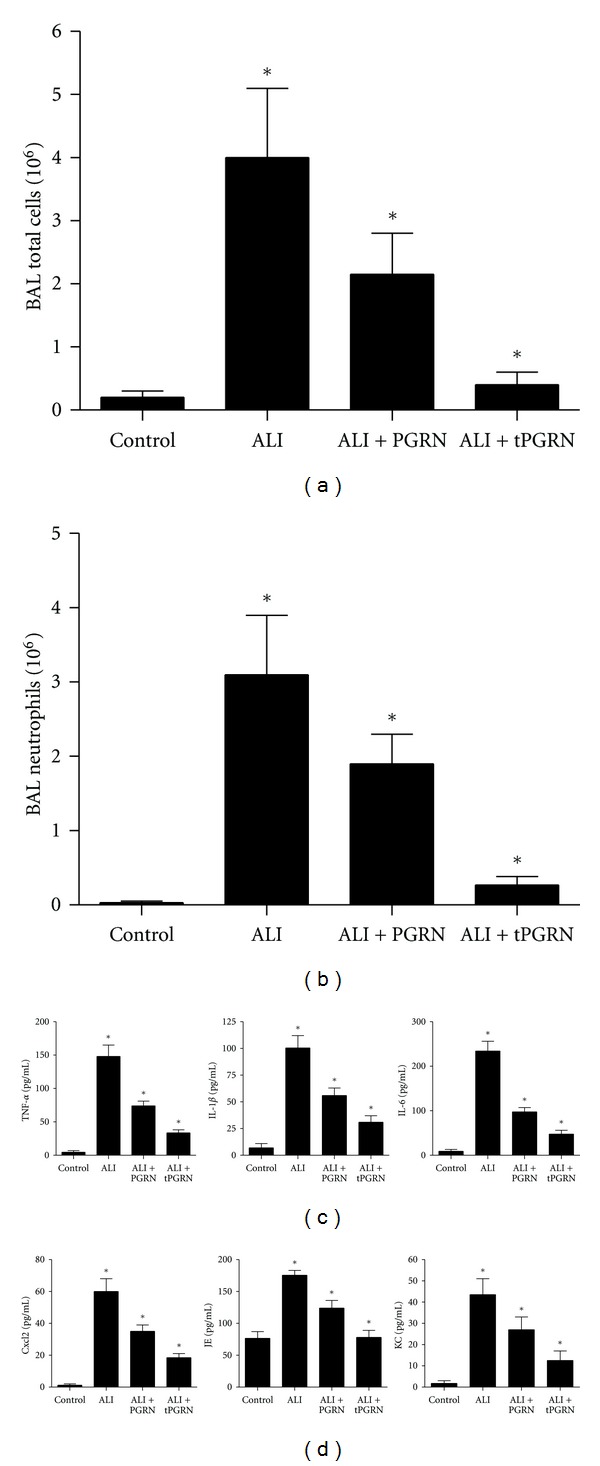
Administration of PGRN attenuated LPS-induced pulmonary inflammation. Groups of mice were challenged with LPS and treated with PGRN 30 min later. The tPGRN represented that PGRN was administered twice at 40 h intervals. (a-b) Total cell (a) and neutrophil (b) counts in BAL fluid were detected to evaluate lung airspace inflammation at day 3 after LPS challenge. (c and d) The indicated proinflammatory cytokines and chemokines in BAL fluid were determined at day 3 after LPS challenge. Data are represented as mean ± standard deviation of one experiment consisting of three replicates. Experiments were performed in triplicate. *P < 0.05.

To investigate the possible mechanism underlying the protective effect of PGRN on LPS-induced ALI, we detected the total cell and neutrophil counts in BAL fluid from mice treated with LPS with or without PGEN. As shown in Figure 3(a), the total inflammatory cell count in the BAL fluid was increased dramatically at day 3 after administration of LPS (*P* < 0.05). We revealed that neutrophils accounted for about 80% of the increased inflammatory cells and was significantly elevated in BAL fluid (Figure 3(b), *P* < 0.05). Notably, we found that administration of PGRN could significantly reduce the total cell and neutrophil counts in BAL fluid (Figures 3(a) and 3(b), *P* < 0.05). When PGRN was administered twice at 40 h intervals, it could further reduce the total cell and neutrophil counts in BAL fluid to a significant lower level (Figures 3(a) and 3(b), *P* < 0.05). 

To further assess the anti-inflammatory effect of PGRN, we further detected the proinflammatory cytokines and chemokines in BAL fluid. We found that proinflammatory cytokines, including TNF-*α*, IL-1*β*, and IL-6, as well as chemokines including Cxcl2, JE (the murine homolog of human CCL2), and KC (the murine homolog of human IL-8), were all significantly elevated in BAL fluid in response to LPS challenge (Figures 3(c) and 3(d), *P* < 0.05). In contrast, administration of PGRN effectively decreased the levels of proinflammatory cytokines and chemokines (Figures 3(c) and 3(d), *P* < 0.05). Consistent to the above findings, administration of PGRN twice at 40 h intervals further reduced the proinflammatory cytokines and chemokines to a significant lower level in BAL fluid (Figures 3(c) and 3(d), *P* < 0.05). 

### 3.4. PGRN Reduced the LPS-Induced Lung Permeability

**Figure 4:**
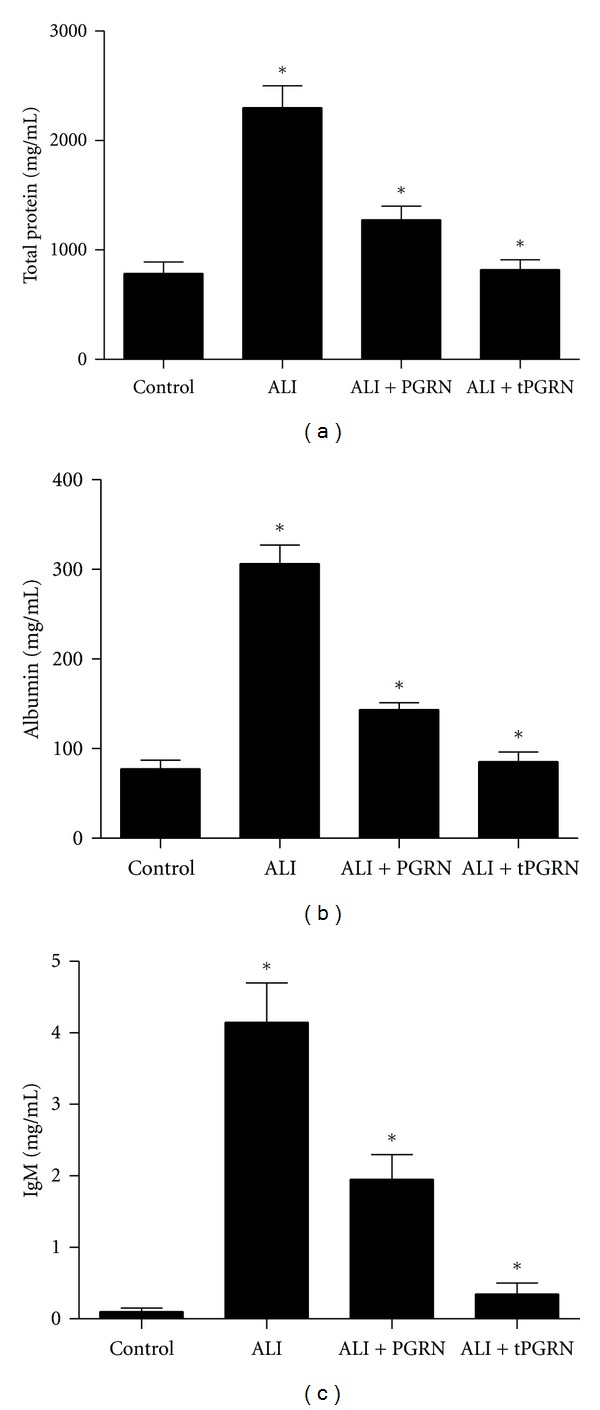
Administration of PGRN reduced the LPS-induced lung permeability. Groups of mice were treated as described above and then the total protein (a), albumin (b), and IgM (c) in BAL fluid were determined at day 3 after LPS challenge. Data are represented as mean ± standard deviation of one experiment consisting of three replicates. Experiments were performed in triplicate. *P < 0.05.

We next determined the concentrations of total protein, albumin, and IgM in BAL fluid to evaluate the integrity of the alveolar-capillary membrane barrier and assess pulmonary vascular leakage as a marker for ALI. As shown in Figures 4(a)–4(c), we found that the levels of total protein, albumin, and IgM in BAL fluid were all significantly increased in mice challenged with LPS compared with that in the control mice (*P* < 0.05). Whereas treatment with PGRN effectively reduced total protein, albumin, and IgM levels (Figures 4(a)–4(c), *P* < 0.05). Notably, administration of PGRN twice at 40 h intervals restored these lung injury indicators to levels similar to the control mice (Figures 4(a)–4(c)). 

### 3.5. PGRN Ameliorated the Histopathology Changes of Lung in LPS-ALI Mice

**Figure 5:**
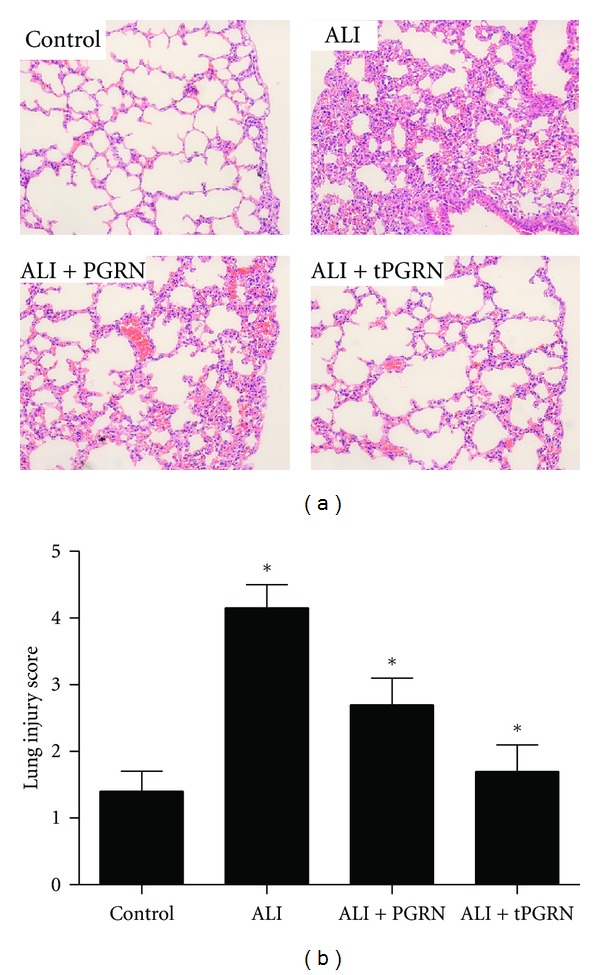
Administration of PGRN ameliorated the histopathology changes of lung in LPS-ALI mice. Groups of mice were treated as described above and then histological evaluation of therapeutic potential of PGRN on LPS-induced lung injury in mice was analyzed at day 3 after LPS challenge. (a) Representative images of hematoxylin and eosin stained lung sections from four experimental groups. (b) Lung injury score was determined. Data are represented as mean ± standard deviation of one experiment consisting of three replicates. *P < 0.05.

To evaluate the potential role of PGRN in the histopathology changes of lung in LPS-induced ALI mice, histological assessment of lung sections 3 days after the administration of LPS with or without treatment was performed. We revealed the marked inflammatory infiltrates, interalveolar septal thickening, and interstitial edema in LPS-induced ALI mice (Figure 5(a)). Administration of PGRN effectively reduced the airspace inflammation, which was more apparent in mice treated with PGRN twice at 40 h intervals (Figure 5(a)). Furthermore, severity of lung injury was also scored using a semiquantitative histopathology score system [5, 21], which evaluates lung injury in four categories: alveolar septae, alveolar hemorrhage, intra-alveolar fibrin, and intra-alveolar infiltrates. We found that treatment with PGRN could significantly reduce lung injury scores, which was more apparent in mice treated with PGRN twice at 40 h intervals (Figure 5(b), *P* < 0.05). 

### 3.6. PGRN/TNFR2 Interaction Was Crucial for the Protective Effect of PGRN on LPS-Induced ALI

**Figure 6:**
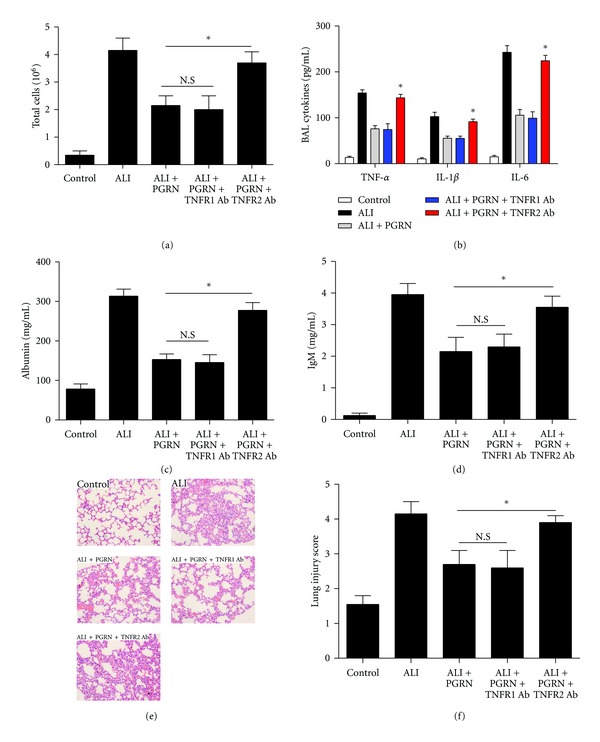
PGRN/TNFR2 interaction was crucial for the protective effect of PGRN on LPS-induced ALI. Groups of mice were pretreated with neutralizing antibodies to TNFR1 or TNFR2, respectively. Twenty-four hours later, the mice were challenged with LPS, followed by administration of PGRN. The total cells (a), proinflammatory cytokines (b), albumin (c), and IgM (d) in BAL fluid was detected at day 3 after LPS challenge. The histological evaluation of lung sections (e) and lung injury score (f) were also analyzed at day 3 after LPS challenge. Data are represented as mean ± standard deviation of one experiment consisting of three replicates. *P < 0.05.

Recent findings suggested that PGRN could bind to TNFR and thus mediate its anti-inflammatory effects in collagen antibody-induced arthritis and collagen-induced arthritis [15]. Therefore, we next assessed the possible role of PGRN/TNFR interaction in the protective effect of PGRN on LPS-induced ALI. Groups of mice were pretreated with neutralizing antibodies to TNFR1 or TNFR2, respectively, and then challenged with LPS with or without PGRN treatment. As shown in Figures 6(a)–6(d), we found that neutralization of TNFR1 had no significant influence on the protective effect of PGRN on the LPS-induced ALI as evidenced by similar levels of total inflammatory cell count, proinflammatory cytokines, albumin, and IgM in BAL fluid. In contrast, blockade of TNFR2 significantly abrogated the protective effect of PGRN on the LPS-induced ALI as evidenced by elevated levels of total inflammatory cell count, proinflammatory cytokines, albumin and IgM in BAL fluid (Figures 6(a)–6(d), *P* < 0.05). Finally, groups of mice were assayed for histological analysis of lung sections. Consistently, we found that blockade of TNFR2 but not TNFR1 could effectively inhibit the protective effect of PGRN on the histopathology changes of lung in LPS-induced ALI mice (Figures 6(e) and 6(f), *P* < 0.05). Similar results were also obtained in mice treated with PGRN twice at 40 h intervals (data not shown). These findings suggested that PGRN/TNFR2 interaction was crucial for the protective effect of PGRN on LPS-induced ALI. 

## 4. Discussion 

ARDS is a complex clinical syndrome that is initiated by injury to the lung, often in the setting of pneumonia or sepsis. Here we carefully evaluated the potential role of PGRN in treatment of ALI using the murine model of LPS-induced ALI. We found that administration of PGRN effectively maintained the body weight and survival of LPS-induced ALI mice. Furthermore, PGRN administration significantly reduced LPS-induced pulmonary inflammation and resulted in remarkable reversal of LPS-induced increases in lung permeability. Moreover, administration of PGRN contributed to a significant reduction of histopathology changes in lung of LPS-induced ALI mice. Our results provided clues for developing PGRN-based therapies to treat with ALI. 

Accumulating data suggested that PGRN played an important role in inflammatory response [15, 23, 24]. Here we evaluated the expression of PGRN protein in BAL fluid of LPS-induced ALI mice. We found that the level of PGRN protein in BAL fluid was significantly downregulated 3 days after LPS challenge in LPS-induced ALI mice. Previous study showed that during inflammation, neutrophils, and macrophages released proteases which digested PGRN into individual 6 kDa granulin units, which were actually proinflammatory and could neutralize the anti-inflammatory effects of intact PGRN [23, 24], which might partly explain the decreased level of PGRN protein in BAL fluid of LPS-induced ALI mice. Consistently, we indeed revealed an elevated expression of granulin, which was the units of PGRN, in BAL fluid in LPS-induced ALI mice. However, the precise mechanism underlies the downregulation of PGRN in BAL fluid of LPS-induced ALI still remains to be elucidated. 

In the present study, we demonstrated that administration of PGRN effectively prevented the development of ALI. Our findings suggested that PGRN was a key regulator of inflammation and exerted an anti-inflammatory effect, which were in line with previous studies [15]. As the half-life time for PGRN is about 40 hours [15], we further performed the second injection of ALI mice with PGRN at 40 h intervals, and found that this strategy resulted in a more apparent reduction of the development of LPS-induced ALI. It should be pointed out that we did not observe any significant effect of PGRN alone on the lung injury of naïve mice in this study (data not shown). Our data strongly suggested that PGRN was an optimistic candidate for the treatment of ALI. However, the LPS-induced model of ALI cannot fully reproduce the complexity of clinical ALI/ARDS in human patients. Therefore, it is necessary to reproduce these findings in more clinically relevant models. Besides, it is important to define the therapeutic window of PGRN intervention for ALI at different dose and time points. In addition, it is also important to explore the possible effect of PGRN administration on host immune response in ALI. The translation of our results into an effective new therapy for ARDS in patients will require, at the very least, that these issues be addressed. 

TNF-*α*/TNFR signaling has received great attention due to its position at the apex of the proinflammatory cytokine cascade and its dominance in the pathogenesis of various disease processes [25–28]. Previous study showed that PGRN could bind to TNFR and then block the TNF-*α* binding to its receptors [15]. In this study, we evaluated the potential role of PGRN/TNFR interaction in the protective effect of PGRN on LPS-induced ALI. We demonstrated that blockade of TNFR2 but not TNFR1 could significantly inhibit the protective effect of PGRN on the LPS-induced ALI. In addition, we found that neutralization of TNFR1 or TNFR2 had no significant effect on the total cell response of ALI mice (data not shown). Our findings were consistent with previous study which showed that TNFR2 seemed to play an important role in ARDS [29]. We presumed two factors that could partly explain this phenomenon. One is that TNFR1 is expressed ubiquitously, whereas TNFR2 expression is tightly regulated and found predominantly in hematopoietic cells [30, 31]. Another is that PGRN exhibited a higher affinity for TNFR2 when compared to TNF-*α* [15]. However, the precise mechanism for the effect of PGRN on the development of LPS-induced ALI undoubtedly needed successive studies. 

## 5. Conclusions

In the present study, we demonstrated a murine model of ALI that administration of PGRN effectively prevented the development of LPS-induced ALI, at least in part, through their interaction with TNFR2. These findings might have potentially important implications for the treatment of ARDS, a clinical syndrome resulting from ALI in human.

## Figures and Tables

**Table 1:** 

Score	Alveolar septae	Alveolar hemorrhage	Intra-alveolar fibrin	Intra-alveolar infiltrations per field
0	All are thin and delicate	No hemorrhage	No intra-alveolar fibrin	Less than 5 intra-alveolar cells
1	Congested alveolar septae in less than 1/3 of the field	Erythrocytes per alveolus in 1 to 5 alveoli	Fibrin strands in less than 1/3 of the field	5 to 10 intra-alveolar cells
2	Congested alveolar septae in 1/3 to 2/3 of the field	At least 5 erythrocytes per alveolus in 5 to 10 alveoli	Fibrin strands in 1/3 to 2/3 of the field	10 to 20 intra-alveolar cells
3	Congested alveolar septae in greater than 2/3 of the field	At least 5 erythrocytes per alveolus in more than 10 alveoli	Fibrin strands in greater than 2/3 of the field	More than 20 intra-alveolar cells
